# Gay, Lesbian, and Bisexual (LGB) peoples' leadership self-effectiveness: The roles of internalized sexual stigma, LGB positive identity, and traditional masculinity

**DOI:** 10.3389/fsoc.2023.1108085

**Published:** 2023-03-10

**Authors:** Marco Salvati, Tunahan Sari, Valerio Pellegrini, Valeria De Cristofaro

**Affiliations:** ^1^Department of Human Sciences, University of Verona, Verona, Italy; ^2^Department of Human and Social Sciences, University of Bergamo, Bergamo, Italy; ^3^Department of Social and Developmental Psychology, Sapienza University of Rome, Rome, Italy

**Keywords:** leadership, gay, lesbian and bisexual people, internalized sexual stigma, positive identity, traditional masculinity and femininity

## Abstract

Grounded in the theoretical frameworks of the minority stress model and the model of positive identity in sexual minority people, the current research contributes to fill a gap in the previous literature, investigating the relationships among leadership self-effectiveness, internalized sexual stigma, positive identity, and adherence to traditional masculinity of gay, lesbian, and bisexual (LGB) individuals. Through a correlational study (*N* = 449), we collected data from 229 gay/bisexual men (51%) and 220 lesbian/bisexual women (49%). We hypothesized that lower internalized sexual stigma, higher LGB positive identity, and higher adherence to traditional masculinity were associated to higher self-perceived effectiveness. The interactive relationships among the variables, including participants' gender, were investigated from an exploratory perspective. The hypotheses were tested through two moderated regression models and the results confirmed that participants with lower internalized sexual stigma and higher LGB positive identity were more likely to perceive themselves as potential effective leaders. Also, the results showed a significant interaction between participants' gender and traditional masculinity score suggesting that high adherence to traditional masculinity was a significant predictor of self-perceived effectiveness only for gay/bisexual men, but not for lesbian/bisexual women. This research contributes to provide both confirmation and novel insights into the key role of relevant factors impacting on LGB people's leadership self-effectiveness, which might contribute to preserve the gay glass ceiling effect. The presence of antidiscrimination policies in organizations not only might reduce reports of discrimination but also enhance LGB employees' positive sense of self, which is a critical aspect to emerge as a leader.

## Introduction

Despite numerous institutional interventions that aim to prevent sexual orientation and gender identity stigmatization and discrimination recently, it is a well-known fact that LGBTQ+ employees still encounter an unequal workplace experience in practice (Mara et al., 2021; Ozbilgin et al., 2022). One of the leading discriminations LGBTQ+ employees face in practice is the “gay glass ceiling effect” in which they report better managerial authority and supervisory skills, but they are not able to attain top managerial positions and even get paid less when compared to heterosexual individuals. Even though LGBTQ+ individuals are more likely to have a longer formal education, they tend to only reach low payment managerial positions because of discrimination, not rather than their different skills or characteristics (Aksoy et al., 2019). Eventually, many LGBTQ+ individuals might feel discouraged when they comprehend emerging as a leader is a challenging process requiring too much effort compared to their heterosexual counterparts (Salvati et al., 2021a).

Understanding the consequences of sexual stigma in the workplace is noteworthy since sexual minority individuals are targeted to be marginalized and discriminated against. Sexual stigma pertains to the negative societal considerations against non-heterosexual behaviors, identities, relationships, or communities (Herek and McLemore, 2013). Collectively, society holds a shared knowledge that all kinds of non-heterosexual behaviors and attractions are not tenable and subject to stigmatization and discrimination. On the other hand, heterosexism is a structural phenomenon in which either every individual is assumed as heterosexual or any recognition of non-heterosexuality is assumed abnormal and justifiable for discriminatory treatment and hostility (Herek et al., 2015). Moreover, sexual stigma might manifest itself as an internalized sexual stigma. Internalized sexual stigma refers to the individual's personal acceptance of sexual stigma constituted by society regardless of gender identity and sexual orientation (Herek and McLemore, 2013; Herek et al., 2015). While heterosexual individual's internalized sexual stigma shows up as negative attitudes toward LGBTQ+ individuals (Herek et al., 2015), LGBTQ+ individual's internalized sexual stigma might be both internal and external (Herek et al., 2015; Sommantico et al., 2018). In other words, LGBTQ+ individuals with high levels of internalized sexual stigma might have negative attitudes not only toward their own gender identity and sexual orientation but also toward other LGBTQ+ individuals.

In organizations where heterosexism is dominant, LGBTQ+ employees perceive career-related barriers based on their gender identity and sexual orientation (Schmidt et al., 2012; Allan et al., 2015). Sexual stigma might have negative impacts on LGBTQ+ employees' career advancement (Fassinger et al., 2010). The scholars also emphasize that LGBTQ+ employees are concerned that their effectiveness and success will be seen as inadequate when they become leaders. Hence, it might be understandable that the more LGBTQ+ employees internalize sexual stigma against gender identity and sexual orientation, the lower self-efficacy they may have. Furthermore, several authors argued that gay and lesbian employees who have disclosed their sexual orientation might be prevented in their career path compared to their LGBTQ+ counterparts who did not (Buser et al., 2015; Dilmaghani, 2018). In their study, Salvati et al. (2021a,b) found similar results that gay employees with higher internalized sexual stigma are less likely to apply for a leadership position because of their sexual orientation. Hence, it has been hypothesized that there is a significant and negative association between internalized sexual stigma and self-perceived effectiveness as a potential leader.

While some LGBTQ+ individuals internalize sexual stigma based on gender identity and sexual orientation, some LGBTQ+ individuals tend to embrace their gender identity and sexual orientation. Minority stress theory conceptualized by Meyer (2003) states that LGBTQ+ individuals with their stigmatized social identities might experience additional stressors based on their gender identity or sexual orientation in addition to job-related stress in heterosexist environments. This internalization process of stigma is a proximal stressor in which LGBTQ+ individuals might have internalized sexual stigma, expect or fear rejection, and try to hide their gender identity and sexual orientation (Meyer, 2003). Similarly, internalized sexual stigma might not only lead to concealment at work but also provoke personal distress (Velez et al., 2013). Hence, having a positive identity might be a protective factor against discrimination and stigmatization for sexual minorities living in heteronormative contexts (Riggle and Rostosky, 2011). Positive LGBTQ+ identity refers to having positive feelings and thoughts while defining yourself as an LGBTQ+ -identified person (Rostosky et al., 2018). Scholars emphasize that having a positive LGBTQ+ identity is not simply equivalent to not having internalized sexual stigma, but a positive LGBTQ+ identity is more of a multi-dimensional process rather than a spectrum (Mohr and Kendra, 2011; Petrocchi et al., 2020). Several studies considered these dimensions that LGBTQ+ individuals hold positive perceptions about the aspects of LGBTQ+ identity including commitment to social justice, sense of belonging to a community, authenticity, self-awareness, and satisfaction in romantic relationships (Rostosky et al., 2010; Riggle et al., 2014; Sung et al., 2015). Having a positive LGBTQ+ identity has been found to be associated with individuals' psychological well-being (Riggle and Rostosky, 2011; Baiocco et al., 2018). In their study, Petrocchi et al. (2020) investigated the effect of having a positive LGBTQ+ identity on the well-being of Italian lesbian women, gay men, and bisexual people. The scholars found out that lesbian and gay participants hold higher levels of self-positive identity perception compared to bisexual people. Moreover, they stated that self-awareness, community, authenticity, and intimacy which are the dimensions of positive LGBTQ+ identity have a significant and positive contribution to the well-being of individuals. Previous research showed that a positive LGBTQ+ identity may act as a strength and resource to overcome the sexual stigma constituted by society and promote resilience for sexual minority groups within different contexts (Vaughan and Rodriguez, 2014). Therefore, we can hypothesize that employees who develop a positive LGB identity would see themselves as more effective in leadership positions, compared to employees with a negative LGB identity.

Social role theory developed by Eagly (1987) not only claims that gender stereotypes form a standard prototype for descriptive roles of men and women but also constitute normative roles that how men and women should behave in certain situations (Coffman, 2014). Hence, men and women might tend to act according to their socially assigned gender roles. The dominant stereotypical view in society is that men are described with masculine and agentic traits such as being competitive and dominant, whereas women are considered to hold more feminine and communal traits such as being warm, compassionate, empathic, and socially oriented (Salvati et al., 2019; Rosca et al., 2020; Kosakowska-Berezecka et al., 2022). According to the role congruity model (Eagly and Karau, 2002), there should be a match between the way how an individual is seen and the traits and behaviors a successful leader should have. Based on social role theory, stereotypes related to women's roles are considered less consistent with leadership positions (Eagly and Wood, 2012). Therefore, women leaders are evaluated as less effective than male leaders (Heilman et al., 2004). Gender stereotypes related to heterosexist ideology also stand upon LGBTQ+ individuals to conform to assigned gender roles. Thus, LGBTQ+ employees may try to cope with the stigmatization and discrimination by trying to fit the social norms and they put much effort to close the gap between how they act and how they are expected to act (Ozbilgin et al., 2022). Previous research found that lesbian women perceived themselves as more masculine than bisexual and straight women (Kachel et al., 2016). In addition to this, straight men also perceived themselves as more masculine than gay men, and most gay men participants perceived themselves as more masculine than feminine (Kachel et al., 2016). Moreover, perceiving oneself to have masculine traits and agentic behaviors are thought to be antecedents of being an effective LGBTQ+ leader (Koenig et al., 2011; De Cristofaro et al., 2020; Salvati et al., 2021a; Shamloo et al., 2022). On the other hand, Fasoli and Hegarty (2020) focused on the impact of sexual orientation vocal cues on heterosexual peoples' evaluations of leadership suitability and employability, founding that lesbian-sounding women were evaluated as less suitable than heterosexual-sounding women, and that the attributions of stereotypical masculinity to lesbian-sounding women were shown to be irrelevant to discrimination. Also, the study by Wang et al. (2022) showed that same-sex leaders with other marginalized identities (i.e., being women) do not suffer a double stigma penalization. Based on social role theory and the role congruity model, we might infer that LGBTQ+ individuals with masculine self-perception are more likely to see themselves as potential effective leaders (Salvati et al., 2021a; Shamloo et al., 2022), even though other studies showed inconsistent results for lesbian women (Fasoli and Hegarty, 2020; Wang et al., 2022).

### The current study and hypotheses

The current study focuses on exploring the leadership self-effectiveness of gay, lesbian, and bisexual (LGB) individuals in a sample which mainly consist of individuals who are from U.S. and U.K. In Western societies, such as the U.S. and U.K., discrimination against sexual minority employees is punished by laws (Mize, 2016; Office for National Statistics, 2017; ILGA Europe, 2022). Nevertheless, several disparities still exist both in U.S. and U.K., like the one that gay men earn less that heterosexual men in the same job position, while lesbian women do not show the same pattern (Aksoy et al., 2019).

In-depth, we investigated the direct and interactive associations of internalized sexual stigma, LGB positive identity, traditional masculinity-femininity, and participants' gender with leadership self-effectiveness. Although the current literature includes studies on how LGBTQ+ individuals' leadership effectiveness is perceived by heterosexual people (Morton, 2017; Clarke and Arnold, 2018; Wang et al., 2022), there are not many studies on how LGBTQ+ individuals evaluate their effectiveness (but see Salvati et al., 2021a). Furthermore, the current literature focuses on how gay male employees are perceived for leadership positions; (Morton, 2017; Clarke and Arnold, 2018; De Cristofaro et al., 2020; Pellegrini et al., 2020; Salvati et al., 2021a), whereas studies considering lesbian women and bisexual individuals are more scarce (Fasoli and Hegarty, 2020; Shamloo et al., 2022; Wang et al., 2022). Therefore, the current study aims at filling the gap by putting emphasis on perceived self-effectiveness and including gay, lesbian, and bisexual participants.

Based on social role theory (Eagly, 1987) and the role congruity model (Eagly and Karau, 2002; Heilman et al., 2004), we expected that high levels of internalized sexual stigma would be associated with low levels of perceived self-effectiveness as a potential leader (Hypothesis 1), whereas having a high positive LGB identity would be associated with high perceived self-effectiveness as a potential leader (Hypothesis 2). Also, we expected that high adherence to traditional masculinity would be associated with high perceived self-effectiveness as a potential leader (Hypothesis 3). Considering the scarcity of literature on the topic, the interactive relationships between the variables are investigated from an exploratory perspective, as well as the interactive relationships with the participants' gender.

## Methods

### Power and sample size

Although our main hypotheses were not focused on interactive effects, we decided to determine the sample size based on a moderated regression research design. This would ensure us to obtain an adequate statistical power for also exploring the presence of potential interactive effects. Thus, we ran an a-priori analysis for a linear multiple regression model (*F*-test family) by setting a small f2 of 0.02, a conventional power of 0.80 and an error probability of 0.05. Given the lack of previous literature on the interested interactive associations, we opted for low expected effect size (Cohen, 1988) in our sample size estimation (Perugini et al., 2018). With one tested coefficient (i.e., the interaction) on a total of three (i.e., two main effects and interaction), the analysis revealed a minimum sample size of 391 participants. The analysis has been performed with G^*^power.

### Participants and procedure

Participants were recruited online during the month of February 2021 through Prolific, a software that allows you to recruit and pay research participants by selecting inclusion criteria. The inclusion criteria were: (a) be a native speaker of English; (b) be at least 18 years old; (c) have a cisgender gender identity; d) have a homosexual or bisexual sexual orientation. Based on the inclusion criteria, 449 participants (229 gay/bisexual men, 51.0%; 220 lesbian/bisexual women, 49.0%) (*M*_*Age*_= 34.27; *SD*_Age_ = 12.48) completed the online questionnaire (See Table 1 for more detailed demographics).

**Table 1:** LGB sample's descriptives (*N* = 449).

**Variable**	***N***	***%***
**Gender**		
Male	229	51%
Female	220	49%
**Sexual orientation**		
Bisexual	10	2.2%
Predominantly homosexual	36	8.0%
Exclusively homosexual	403	89.8%
**Nationality**		
U.S.	151	33.6%
U.K.	282	62.8%
Other	16	3.6%
**Educational level**		
Primary school diploma	1	0.2%
Middle school diploma	5	1.1%
High school diploma	140	31.2%
Bachelor's degree	212	47.2%
Master's degree	73	16.3%
PhD or higher specialization	18	4.0%
**Ethnicity**		
Asian	24	5.3%
Black	16	3.6%
Latino	13	2.9%
White/caucasian	381	84.9%
Other	14	3.1%

There was a compensation of £ 5.00 per hour. The time to complete the online questionnaire hosted on Qualtrics was ~10 min. Before starting the questionnaire, participants were told that they were about to participate in research on leadership. Before taking part in the research, everyone read and signed informed consent online which adhered to the revised Declaration of Helsinki (World Medical Association, 2007) and was approved by the Research Ethics Committee of the Department of Social and Developmental Psychology (Removed for blind revision). At the end of the questionnaire, participants were thanked for their participation and sent back to the Prolific site for the compensation.

### Measures

#### Socio-demographics

Participants were asked to indicate their gender (1 = *Male*; 2 = *Female*; 3 = *Other*), age, sexual orientation (1 = *Exclusively heterosexual*; 2 = *Predominantly heterosexual*; 3 = *Bisexual*; 4 = *Predominantly homosexual*; 5 = *Exclusively homosexual*), nationality, ethnicity, and educational level (1 = *Primary school diploma*; 2 = *Middle school diploma*; 3 = *High school diploma*; 4 = *Bachelor's degree*; 5 = *Master's Degree*, 6 = *PhD or higher specialization*).

#### Internalized sexual stigma

ISS was measured by administering the three-item subscale ‘internalized homonegativity' of the Lesbian, Gay, and Bisexual Identity Scale (LGBIS, Mohr and Kendra, 2011). Participants responded on a 6-point Likert scale, ranging from 1 = *Strongly Disagree* to 6 = *Strongly agree*. The three items were: “*If it were possible, I would choose to be straight”, “I wish I were heterosexual”, “I believe it is unfair that I am attracted to people of the same sex”*. The ISS score was calculated through the average of the three items, so that higher scores corresponded to higher levels of ISS. In the current study, Cronbach's Alpha was 0.89. (Cronbach's Alpha ranged between 0.86 and 0.93 in the original validation study by Mohr and Kendra, 2011).

#### LGB positive identity

The three-item subscale ‘Identity Affirmation' of the LGBIS (Mohr and Kendra, 2011) was provided to measure participants' positive identity regarding their sexual identity. Participants responded on a 6-point Likert scale, ranging from 1 = *Strongly Disagree* to 6 = *Strongly agree*. The three items were: “*I am glad to be an LGB person*”, “*I'm proud to be part of the LGB community*”, “*I am proud to be LGB*”. The total score was calculated through the average of the three items, so that higher scores corresponded to higher levels of LGB positive identity. In the current study, Cronbach's Alpha was 0.91 (Cronbach's Alpha ranged between 0.89 and 0.94 in the original validation study by Mohr and Kendra, 2011).

#### Traditional masculinity-femininity

Participants were administered the six-item TMF Scale (Kachel et al., 2016), which required to attribute a score from 1 = *Very Feminine*, to 7 = *Very Masculine* to six incomplete sentences such as: “*I consider myself as…*”, “*Traditionally, my interests would be considered as…*”, “*Traditionally, my attitudes and beliefs would be considered as…*”. The total score was calculated through the average of the six items, so that high scores corresponded to higher levels of traditional masculinity, whereas low scores corresponded to higher levels of traditional femininity. In the current study, Cronbach's Alpha was 0.87. (Cronbach's Alpha was 0.94 in the original validation study by Kachel et al., 2016).

#### Leadership self effectiveness

Participants completed the ten-item scale of leadership effectiveness by Hais et al. (1997), which was readapted and already used in previous studies on gay (De Cristofaro et al., 2020; Pellegrini et al., 2020; Salvati et al., 2021a) and lesbian leadership (Shamloo et al., 2022). Example items were: “*I have the qualities for being a good leader*”, “*I would be an effective leader*”, and ‘*I would be willing to endorse a leader like me*”. This tool detects leadership self-effectiveness, without referring to a specific leadership context. The total score was calculated through the average of the ten items, so that higher scores corresponded to higher levels of self-effectiveness as potential leader. In the current study, Cronbach's Alpha was 0.96 (Cronbach's Alpha was 0.88 in the original validation study by Hais et al., 1997).

### Statistical analyses

Before proceeding to test our hypotheses, we conducted preliminary analyses investigating correlations, kurtosis and skewness statistics among the measure collected (Table 2). Such analyses allowed to explore assumptions of normality and multicollinearity in our data. Subsequently, two moderated regression models tested our research hypotheses.

**Table 2:** Correlations and descriptives.

	**Gender**	**Age**	**Education**	**LGB PI**	**ISS**	**TMF**	**EFF**
Gender	1						
Age	−0.17^**^	1					
Education	−0.06	0.15^**^	1				
LGB PI	0.18^**^	−0.15^**^	−0.07	1			
ISS	−0.05	−0.03	0.06	−0.61^**^	1		
TMF	−0.40^**^	0.30^**^	0.05	−0.16^**^	0.02	1	
EFF	−0.06	0.11^*^	0.09	0.19^**^	−0.15^**^	0.15^**^	1
M	-	34.28	-	4.70	1.67	4.16	3.50
SD	-	12.48	-	1.30	1.13	1.08	0.97
Skewness	-	1.04	-	−0.95	2.01	−0.25	−0.63
Kurtosis	-	0.53	-	0.14	3.58	0.24	−0.17

* p < 0.05;

** p < 0.01.

Gender: 1, Male (Gay and Bisexual Men); 2, Female (Lesbian and Bisexual Women); LGB PI, LGB Positive Identity; ISS, Internalized Sexual Stigma; TMF, Traditional Masculinity-Femininity Scale: High scores correspond to high traditional masculinity; EFF, Leadership Self-Effectiveness.

Specifically, in the first moderated regression model, ISS was entered as predictor (X), EFF as dependent variable (Y), and TMF (M1) and participants' gender (M2) as moderators (Model 2 by vers. 4.0 of PROCESS of SPSS; Hayes, 2017). In the second moderated regression model, LGB PI was entered as predictor (X), EFF as dependent variable (Y), and TMF (M1) and participants' gender (M2) as moderators (Model 2 by vers. 4.0 of PROCESS macro of SPSS; Hayes, 2017).

As additional exploratory analysis, in order to explore the interactive effect between participants' TMF and gender, we have run an additional moderated regression model (Model 1 by vers. 4.0 of PROCESS macro of SPSS; Hayes, 2017) where participants' TMF and gender were the predictor (X) and moderator (M) respectively, whereas ISS and LGB PI were included as covariates.

## Results

### Correlation and preliminary analyses

Preliminary analyses showed that all the measures confirm normality assumptions, indeed all the absolute skewness and kurtosis values are lower than 3 and 8, respectively (Kline, 2015). Also, correlation results indicated that multicollinearity was not an issue, showing that all the correlations are below the threshold of |0.80| (Field, 2009). Descriptives by gender are shown in Table 3.

**Table 3:** Descriptive statistics by gender.

**Variable**	**Gay/bisexual men** ***N*** = **229**		**Lesbian/bisexual women** ***N*** = **220**	
	***M***	***SD***	***M***	***SD***
Age	36.34^a^	12.96	32.13^b^	11.60
Education	3.95^a^	0.06	3.85^a^	0.06
LGB PI	4.47^a^	1.30	4.93^b^	1.25
ISS	1.73^a^	1.14	1.60^a^	1.12
TMF	4.58^a^	0.93	3.73^b^	1.05
EFF	3.56^a^	1.00	3.44^a^	0.95

Statistical significant gender differences are showed through different letter in superscript to the mean values.

LGB PI, LGB Positive Identity; ISS, Internalized Sexual Stigma; TMF, Traditional Masculinity-Femininity Scale: High scores correspond to high traditional masculinity; EFF, Leadership Self-Effectiveness.

The correlation results are in line with our expectations, giving first support to our hypotheses. Indeed, EFF showed e negative association with ISS, *r* = −0.15, *p* < 0.01, with a low effect size (Cohen, 1988), indicating that LGB participants with high ISS are less likely to perceive themselves as effective leaders. On the contrary, the results indicated that EFF was positively associated with LGB Positive Identity, *r* = 0.19, *p* < 0.01, with a low-medium effect size (Cohen, 1988), suggesting that LGB people with a more positive identity tend to report high levels of EFF. Also, es expected EFF showed a positive association with TMF, *r* = 0.15, *p* < 0.01 with a low effect size (Cohen, 1988), indicating that LGB persons' traditional masculinity is associated to high score of leadership self-effectiveness.

### Moderated regression model with internalized sexual stigma

Overall, the model explained a significant proportion of variance, *R*^2^ = 5.19%, *F*_(5, 443)_ = 4.85, *p* < 0.001. Specifically, as expected ISS was negatively associated with EFF, ß = −0.15, *se* = 0.05, *t* = −2.88, *p* = 0.001 with no interaction neither with TMF, ß = −0.01, *se* =0.05, *t* = −0.18, *p* = 0.859, nor with participants' gender, ß = 0.08, *se* = 0.05, *t* = 1.52, *p* = 0.129, indicating that high scores in internalized sexual stigma are associated to low self-perceived leadership effectiveness, independently by participants' traditional masculinity and gender, confirming our hypothesis 1. As expected TMF was positively associated with EFF, ß = 0.14, *se* =0.05, *t* = 2.88, *p* = 0.004, showing that higher score in traditional masculinity is related to higher self-perceived effectiveness, supporting our hypothesis 3. The direct effect of participants' gender on EFF was not significant, ß= −0.03, *se* =0.05, *t* = −0.65, *p* =0.517, showing that gay/bisexual men and lesbian/bisexual women did not report significant statistical differences in leadership self-effectiveness.

### Moderated regression model with LGB positive identity

Overall, the model explained a significant proportion of variance, *R*^2^ = 7.17%, *F*_(5, 443)_ = 6.84, *p* < 0.001. Specifically, as expected LGB PI was positively associated with EFF, ß = 0.22, *se* =0.05, *t* = 4.74, *p* < 0.001, with no interaction neither with TMF, ß = −0.02, *se* =0.05, *t* = −0.33, *p* =0.743, nor with participants' gender, ß = −0.05, *se* =0.05, *t* = −1.03, *p* = 0.302, indicating that high scores in LGB positive identity are associated to high self-perceived effectiveness, independently by participants' traditional masculinity and gender, confirming our hypothesis 2. As in the first model, TMF was positively associated with EFF, ß =0.17, *se* =0.05, *t* = 3.42, *p* < 0.001, showing that higher score in traditional masculinity is related to higher self-perceived effectiveness, supporting our hypothesis 3. The direct effect of participants' gender on EFF was not significant, ß = −0.03, *se* =0.05, *t* = −0.62, *p* =0.537, showing that gay/bisexual men and lesbian/bisexual women did not report significant statistical differences in leadership self-effectiveness.

### Additional exploratory analysis

**Figure 1:**
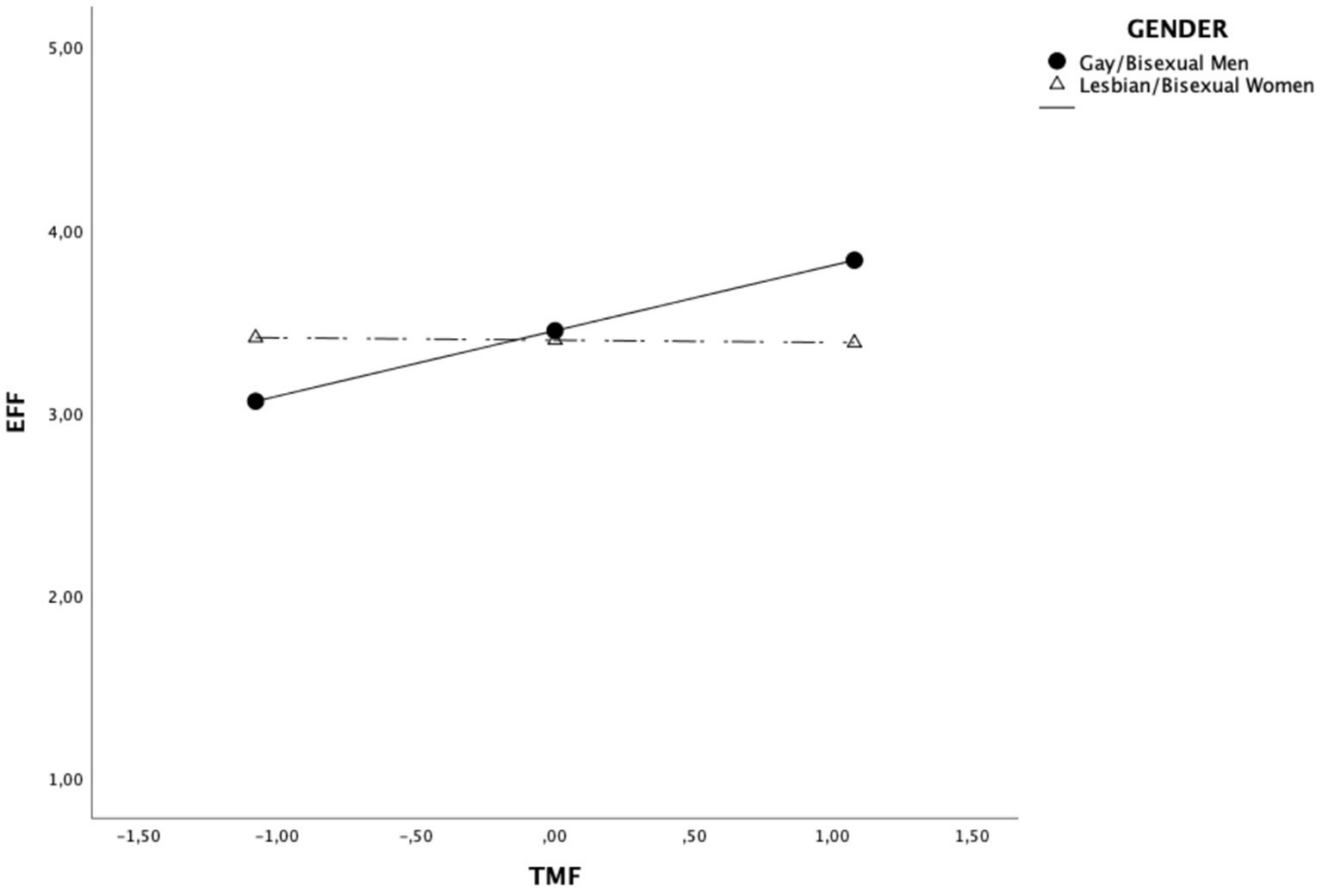
Simple slopes analyses of the interaction TMFxGender on EFF. EFF, Self-perceived leadership effectiveness; TMF, Traditional Masculinity-Femininity Scale: Higher scores correspond to higher traditional masculinity.

On the one hand, such analysis confirmed the results of the previous main analyses showing that TMF was positively associated with EFF, ß = 0.19, *se* =0.05, *t* = 3.90, *p* < 0.001, and that the direct effect of participants' gender on EFF was not significant, ß = −0.03, *se* =0.05, *t* = −0.54, *p* = 0.592. On the other hand, such a model allowed us to show that the direct effect of TMF on EFF was qualified by the interaction with the participants' gender, ß = −0.20, *se* = 0.05, *t* = −4.14, *p* < 0.001. Specifically, simple slope analyses clarified that high scores in traditional masculinity are associated to higher self-perceived leadership effectiveness only in gay/bisexual men, ß = 0.39, *se* = 0.07, *t* = 5.35, *p* < 0.001, but not in lesbian/bisexual women, *B* = −0.01, *se* =0.07, *t* = −0.21, *p* = 0.830 (Figure 1).

## Discussion

The present study aimed to make significant contributions to the studies on leadership effectiveness by investigating the self-perceptions of gay, lesbian, and bisexual individuals. For this purpose, we investigated the impacts of internalized sexual stigma, LGB positive identity, and adherence to traditional masculinity on leadership self-effectiveness. Additionally, whether and how participants' gender and traditional masculinity affect the relationship between internalized sexual stigma, LGB positive identity, and leadership self-effectiveness was examined.

Our first model showed that internalized sexual stigma has a significant and negative impact on leadership self-effectiveness. Thus, our first hypothesis indicating that the more individuals internalize the sexual stigma, the less they perceived themselves as a potential effective leader was supported. This result is consistent with the previous studies emphasizing that embracing the sexual stigma about your sexuality might influence the way how you perceive yourself as a potential leader (Fassinger et al., 2010; Salvati et al., 2021a). This might be partially explained by the fact that LGB people with high internalized sexual stigma tend to enact heteronormative practices through the adhenre to traditional masculinity and femininity and through the rejection of behaviors which are not considered gender role conforming in order to consider themselves worthy of leadership positions (Eagly, 1987; Eagly and Karau, 2002; Heilman et al., 2004; Salvati et al., 2018). By doing this, they can also hinder their career development.

The second hypothesis which defended that LGB positive identity has a significant and positive contribution to leadership self-effectiveness was also supported in the second model. Indeed, our findings revealed that when individuals are glad and proud to be an LGB person and about their presence in the LGB community, they are more likely to perceive themselves as potential effective leaders. Our result is in line with Riggle and Rostosky's (2011) assumptions that having a positive LGB identity perception might help individuals to boost their self-esteem in their working lives and their motivation to reach higher positions. Moreover, Riggle and Rostosky (2011) also argue that when LGB individuals achieve this goal, they might not only become efficient leaders but also role models for others by showing them the vital importance of embracing their LGB identity. Our result supports these assumptions empirically and extends previous research findings which were focused on internalized sexual stigma exclusively (Salvati et al., 2021a).

Our third hypothesis which predicted that traditional masculinity would have a significant and positive contribution to leadership self-effectiveness was also supported. In other words, the more LGB individuals perceive and describe themselves as traditionally masculine, the more their self-perception of becoming effective leaders strengthens. Our finding is in line with previous countless studies on leadership addressing that holding masculine traits is a strong antecedent of becoming an effective leader (Liberman and Golom, 2015; De Cristofaro et al., 2020; Salvati et al., 2021a). However, our results highlighted that high traditional masculinity is a significant predictor of self-perceived effectiveness only for gay/bisexual men, but not for lesbian/bisexual women. Even though LGB individuals struggle to fit in with normative expectations at work and in life, gay/bisexual men who violate traditional gender roles are inclined to face more stigmatization and prejudices, compared to gay/bisexual men who conform to traditional gender roles (Steffens et al., 2015; Salvati et al., 2021b). It might be reasonable to underline that gay/bisexual men are more expected to adhere to traditional masculine roles to fit in and be accepted by others (Vandello and Bosson, 2013; Bosson et al., 2021). This might be one reason in our study why gay/bisexual participants' belief about holding more masculine traits led them to evaluate themselves as more effective potential leaders, compared to lesbian/bisexual women. On the other hand, another possible explanation might be those lesbian/bisexual women are expected to have both masculine and feminine traits for becoming effective leaders (Niedlich and Steffens, 2015; Shamloo et al., 2022). By doing this, they would meet the most dominant criteria of becoming a good leader which is “having masculine traits” and having feminine traits would come up as a coping strategy not to break gender roles (Fassinger et al., 2010; Niedlich and Steffens, 2015). The study by Kachel et al. (2016) could support this by showing that lesbian and bisexual women consider themselves less masculine than gay and bisexual men.

### Practice implications

This research contributes to providing both confirmation and novel insights into the key role of relevant factors impacting on LGB people's leadership self-effectiveness such as internalized sexual stigma, positive LGB identity, and traditional masculinity, which might contribute to preserving the gay glass ceiling effect. Drawing on the minority stress theory developed by Meyer (2003), we might assume that LGBTQ+ individuals are at risk of experiencing stigmatization, marginalization, and discrimination in heterosexist institutional settings. They are not only exposed to job-related stressors but also to minority-specific stressors. On the other hand, this heterosexist working environment might lead LGBTQ+ individuals to internalize sexual stigma. Thus, it is very crucial to develop institutional interventions to prevent discrimination, marginalization, and stigmatization against sexual minorities. The presence of antidiscrimination policies in organizations not only reduces reports of discrimination (Barron and Hebl, 2013) but also enhances LGBTQ+ employees' positive sense of self (Riggle et al., 2010). As we mentioned before, it is very critical having a positive sense of self for LGBTQ+ employees to emerge as a leader. Furthermore, organizations should also put forward inclusivity training and diversity management programs. LGBTQ+ employees are less likely to report interpersonal discrimination when their organization develops antidiscrimination policies and diversity training that consider sexual minorities (Button, 2001). Organizations that give importance to diversity and inclusion might prevent violence against LGBTQ+ employees by developing such practical strategies. Moreover, these organizations might even help to prevent LGBTQ+ individuals from internalizing assigned gender roles and to develop a positive LGBTQ+ identity. LGBTQ+ employees with low internalized sexual stigma and high positive identity perception might feel more confident to attain higher positions. Of course, we do not believe nor that “masculinity” should be taught to lesbian and bisexual women, nor that gay and bisexual men should train certain behaviors in line with traditional masculinity norms. In our opinion, being aware of these relationships is in itself already an element that contributes to the awareness and understanding the phenomenon of the gay ceiling effect. In our opinion, it would be better to intervene on the reduction of internalized sexual stigma and on promoting a positive LGB identity. Therefore, organizations might get benefit from these individuals and not waste these talents.

## Limitations and future research directions

The current study is not without limitations. Firstly, the correlational nature of our data does not allow us to infer causal-effect relationships. Future studies might corroborate and extend our preliminary findings by conducting experimental studies (i.e., by manipulating participants' masculinity and femininity through a fictitious score on a test; Salvati et al., 2021b). On a related methodological note, we used self-reports to measure our variables, whereas future studies might focus on using objective measures of leadership effectiveness. Moreover, qualitative studies might be performed to get a deeper understanding of LGBTQ+ individuals' beliefs and experiences about their leadership experiences and self-effectiveness perception. Thirdly, we did not focus on a specific job context, but we asked our LGB participants to evaluate themselves as a potential effective leader in general. Future studies might consider specific job contexts which are stereotypically perceived as more “masculine” or “feminine”. Indeed, based on gender stereotypes, some occupations could be associated with women while others with men (Heilman, 1983; Eagly, 1987). Thus, based on the lack-of-fit-model (Heilman, 1983) and the gender stereotypes affecting gay and lesbian individuals, heterosexual people and LGB individuals themselves might perceive a gay man and a lesbian woman as more suitable for a leadership position in a stereotypical female-typed or masculine-type occupation, respectively (Clarke and Arnold, 2018; Pellegrini et al., 2020).

A further limitation might be the choice to use moderated regression models, rather than path analysis models or multigroup analyses, which would allow to analyze all the direct and the interactive effects jointly, including both internalized sexual stigma and LGB positive identity as two main predictors simultaneously. The current hypotheses and sample size did not provide a reason to formulate models with more interaction terms, but future studies might deepen the relationships among the variables tested, in order to enrich the picture. Lastly, although our study extends the results of previous literature by involving lesbian/bisexual women too, however, the generalizability of our findings is still limited because they are not applicable to the whole LGBTQ+ people. Future studies might include employees with other gender identities and sexual orientations in order to avoid perpetuating their invisibility also within the LGBTQ+ community since they are one of the pioneer subjects of the glass ceiling effect (Salvati and Koc, 2022).

## Conclusion

The current research has as an innovative strength the focus on the positive dimension of LGB identity which can, unlike internalized sexual stigma, positively impact on leadership effectiveness of LGB people. Such an aspect is relevant in terms of application and intervention implications, suggesting to focus not only on programs aimed at reducing internalized sexual stigma in LGBTQ+ people but also and above all aimed at developing a positive LGB identity and feelings of pride. At the same time, this research supported the previous results showing that adhering to traditional masculinity is still a key factor for gay/bisexual men (but not for lesbian/bisexual women) which affect their self-percevived leadership effectiveness. Future studies might consider the multidimensional aspects that characterize LGB positive identity in order to investigate what are the ones mainly related to the leadership self-effectiveness, and consequently having a more complete and articulated view of the various relationships.

## Data availability statement

The raw data supporting the conclusions of this article will be made available by the authors, without undue reservation.

## Ethics statement

The studies involving human participants were reviewed and approved by Department of Social and Developmental Psychology, Sapienza University of Rome. The patients/participants provided their written informed consent to participate in this study.

## Author contributions

Conceptualization and methodology: MS, VP, and VD. Formal analysis: VP. Investigation and supervision and project administration: MS and VD. Resources and data curation: MS. Writing—original draft: TS and MS. Writing—review and editing: TS, MS, and VD. All authors have read and agreed to the published version of the manuscript.
